# Morphometric analysis of foramen ovale, foramen spinosum, and foramen rotundum of human skull using computed tomography scan: a cross-sectional study

**DOI:** 10.1097/MS9.0000000000000609

**Published:** 2023-04-11

**Authors:** Rakshya Bhattarai, Sagar Panthi, Gopal K. Yadav, Siddhartha Bhandari, Rochana Acharya, Ananta Sharma, Pratima K. Shah, Sarun Koirala, Manoj Bhattarai, Mukesh K. Gupta, Bhawani Khanal

**Affiliations:** Departments of a Radiodiagnosis and Imaging; b Surgery; c Internal Medicine; d Anatomy, BP Koirala Institute of Health Sciences, Dharan, Nepal; e Department of Surgery, Kist Medical College and Teaching Hospital, Lalitpur; f Department of Radiodiagnosis and Imaging, Maharajgunj Medical Campus, Institute of Medicine, Kathmandu, Nepal

**Keywords:** human skull, morphometric analysis, objective measurement, sizes of foramina

## Abstract

There is limited literature of objective assessments of foramina of skull base using computed tomography (CT) scan. This study was carried out to analyze the dimensions of foramen ovale (FO), foramen spinosum (FS), and foramen rotundum (FR) using CT scan imaging of the human skull and their associations with sex, age, and laterality of the body.

**Materials and methods:**

A cross-sectional study was carried out in the Department of Radiodiagnosis and Imaging at BP Koirala Institute of Health Sciences (BPKIHS), Nepal using a purposive sampling method. We included 96 adult patients (≥18 years) who underwent CT scan of the head for any clinical indications. All those participants below 18 years, inadequate visualization or erosions of skull base foramina, and/or not consenting were excluded. Appropriate statistical calculations were done using the statistical package for social sciences (SPSS), version 21. The *P*-value of less than 0.05 was considered statistically significant.

**Results:**

The mean length, width, and area of FO was 7.79±1.10 mm, 3.68±0.64 mm, and 22.80±6.18 mm^2^, respectively. The mean length, width, and area of FS was 2.38±0.36 mm, 1.94±0.30 mm, and 3.69±0.95 mm^2^, respectively. Similarly, the mean height, width, and area of FR was 2.41±0.49 mm, 2.40±0.55 mm, and 4.58±1.49 mm^2^, respectively. The male participants had statistically significant higher mean dimensions of FO and FS (*P*<0.05) than the female participants. There were statistically insignificant correlations of dimensions of these foramina with age and between the left and right side of each foraminal dimensions (*P*>0.05).

**Conclusions:**

The sex-based difference in dimensions of FO and FS should be clinically considered in evaluating the pathology of these foramina. However, further studies using objective assessment of foraminal dimensions are required to draw obvious inferences.

## Introduction

Highlights

- Evaluation of skull base foramina is an important part of diagnostic medicine, especially relevant to anatomists, neuro/head, neck surgeons, and radiologists.
- This study objectively assessed the dimensions of three important foramina of the human skull viz., foramen ovale (FO), foramen spinosum (FS), and foramen rotundum (FR) using computed tomography (CT) scan.
- The male participants had statistically significant higher mean dimensions of FO and FS than the female participants.
- There were statistically insignificant correlations of dimensions of these foramina with age and between left and right side of each foraminal dimensions of FO, FS, and FR.

The FO, FS, and FR are located in the greater wing of the sphenoid bone^1^. The FO is more often irregular in shape with the average maximal and minimal lengths in the adult being 7.48 and 4.17 mm, respectively, and transmits the mandibular nerve (V3), the accessory meningeal artery, the lesser superficial petrosal nerve, and the emissary veins^1^,^2^. The FS is mostly round in shape with an average adult diameter of 2.63 mm and transmits the middle meningeal vessels from the infratemporal to the middle cranial fossa^1^. The FR is round with an average adult diameter of 3.55 mm and transmits the second (maxillary) division of the fifth cranial nerve^1^,^3^. Many normal variants of these foramina have been well described in the early anatomic and radiologic literature^4^. The inequality of size of FO, FS, and FR was noted in 30.89%, 16.26%, and 0%, respectively in the same study of Ginsberg *et al.* 4. The CT scan remains the modality of choice in defining the bony anatomy of the skull base^5^.

There is limited existing data on the symmetry and diameter of skull base foramina on CT, and determination of foramen widening is typically subjective^6^. Evaluation of skull base foramina is becoming an important part of diagnostic medicine, especially relevant to anatomists, neuro/head and neck surgeons, and radiologists. Therefore, this study was carried out with the objective to analyze the dimensions of FO, FS, and FR using CT scan imaging of the human skull and their associations with sex, age, and laterality of the body.

## Materials and methods

### Study design, sample size, and sampling technique

We carried out a cross-sectional study in the Department of Radiodiagnosis and Imaging BP Koirala Institute of Health Sciences (BPKIHS), Nepal. According to a study by Berlis *et al.* 7, CT measured mean breadth of the FR was reported to be 3.11±0.78 mm (mean±SD). Based on this study, the sample size for our study was calculated using the one-sample mean formula at 95% CI with 5% permissible error.


$$n\;=\;Z^{2}\sigma^{2}/L^{2},$$

where, *n*=sample size,

*Z* at 95% CI=1.96,

σ (SD)=0.78, and

*L*=permissible error (i.e. 5% of mean)=0.156.

Therefore, $n\;=\;{\left(1.96\right)}^{2}\;\times \;{\left(0.78\right)}^{2}/{\left(0.156\right)}^{2}\;=96.$

The purposive sampling method was adopted for the collection of the study samples. We included 96 adult patients (≥18 years) who underwent a CT scan of the head for any clinical indication (except for any pathology involving these foramina) over the period of 6 months (7 October 2019–6 April 2020) and who had optimal visualization of middle cranial fossa foramen in bone window settings. All those participants with age below 18 years, poor or inadequate visualization of skull base foramina, skull base fracture, evidence of bone erosion, or destruction affecting the margins of foramina, history of skull base surgery, and/or not consenting to participate were excluded from the study. This work has been carried out in accordance with the STROCSS criteria^8^.

### Measurements of foramina and their area

**Figure 1:**
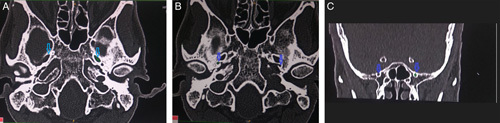
(A) Axial computed tomography image of the skull base (bone window) showing a method of measurement of foramen ovale (arrows), with calipers placed on length and width of left foramen ovale (solid lines). (B) Axial computed tomography image of the skull base (bone window) showing a method of measurement of foramen spinosum (arrows), with calipers placed on length and width of right foramen spinosum (solid lines). (C) Reformatted coronal computed tomography image of the skull base (bone window) showing a method of measurement of foramen rotundum (arrows), with calipers placed on the height and width of left foramen rotundum (solid lines).

All the CT examinations were performed with standard protocol on a 16-slice multidetector CT scanner available in the department (NeuViz 16; Neusoft) as per the pathology/clinical indication of the participants. Volumetric acquisition of image data was obtained and the axial slice generated in the bone window was reconstructed by postreconstruction techniques into thinner slices of 0.625 mm. Three foramina (ovale, spinosum, and rotundum) of middle cranial fossa on each side, that is, a total of six foramina, were viewed in thin image sections in bone window settings (4000ww/400wl) after zooming in the area of interest. Each foramen was defined as a round or oval structure with a sclerotic osseous rim and a lucent center identified on at least two adjacent sections on expected anatomical locations for each of these structures. The FO and FS were measured in axial sections of the skull. The FR was viewed in coronal sections obtained via multiplanar reconstruction. The size of each of the foramina was measured via the electronic caliper in those sections where the foramen was adequately visualized. The maximum anteroposterior diameter (length) and transverse diameter (width) on axial sections were recorded for FO and FS (Figs. 1A and B). The maximum craniocaudal diameter (height) and transverse diameter (width) on the coronal section were recorded for FR (Fig. 1C). The three measurements of the same dimensions were made by three different radiologists and the average was taken as the final value. After recording the respective diameters of each of the foramina, calculation of the area of each of these foramina was done using the standard formula for the area of an ellipse, that is,


$$\mathrm{Area}\;=\;\pi D_{1}D_{2}\;/4,$$

where, *D*_1_=length of FO/FS, and height of FR,

*D*_2_= width of FO/FS/FR.

### Data entry and analysis

The data collected were tabulated in Microsoft Excel 2019, v16.0 (Microsoft) and analysis was carried out using the statistical package for social sciences (SPSS), version 11.5, IBM SPSS, v21 (IBM Corp.). The descriptive statistics like mean, SD, and range were used to present data like diameters and surface areas of each of the foramen. The distribution of data was confirmed by Kolmogorov–Smirnov test before using parametric tests. For comparing the mean of foramen sizes according to body side (left vs. right), paired sample *t*-test was used. An independent sample *t-*test was used to compare the mean of foramen sizes in males and females. Pearson’s correlation test was used to assess the correlation of foramen size with age. The *P*-value of less than 0.05 was considered statistically significant.

## Results

Of 96 patients, two-third of the participants (65, 67.71%) were male. The male-to-female ratio was 2.1:1. The age of the participants ranged from 19 to 89 years with the mean age being 47.28±18.86 years. Of the total participants, two-fifth of the participants (41, 42.71%) belonged to the age group 19 to 39 years (Table 1).

**Table 1:** Background characteristics of the study participants

Variables	*n* (%)
Sex	
Male	65 (67.71)
Female	31 (32.29)
Age (years)	
Mean±SD (minimum–maximum)	47.28±18.86 (19–89)
19–39	41 (42.71)
40–59	27 (28.13)
≥60	28 (29.17)

On averaging the measurements of right and left side, the overall mean±SD for length, width, and area of FO was 7.79±1.10 mm, 3.68±0.64 mm, and 22.80±6.18 mm^2^, respectively. The overall mean±SD for its length, width, and area of FS was 2.38±0.36 mm, 1.94±0.30 mm, and 3.69±0.95 mm^2^, respectively. Similarly, the overall mean±SD for the height, width, and area of FR was 2.41±0.49 mm, 2.40±0.55 mm, and 4.58±1.49 mm^2^, respectively (Table 2).

**Table 2:** Overall dimensions of foramina of the study participants (n=96×2)

Foramen and its dimensions	Mean±SD	Minimum	Maximum
Ovale			
Length (mm)	7.79±1.10	5.95	10.30
Width (mm)	3.68±0.64	2.30	5.45
Area (mm^2^)	22.80±6.18	11.10	41.16
Spinosum			
Length (mm)	2.38±0.36	1.25	3.15
Width (mm)	1.94±0.30	1.05	2.65
Area (mm^2^)	3.69±0.95	1.16	6.26
Rotundum			
Height (mm)	2.41±0.49	1.40	3.90
Width (mm)	2.40±0.55	1.05	4.05
Area (mm^2^)	4.58±1.49	1.23	9.86

The male participants had statistically significant higher mean values of length, width, and area of FO and FS (*P*<0.05) than the female participants. However, despite higher mean values of height, width, and area of FR in male participants than female participants, they are not statistically significant (*P*>0.05) (Table 3).

**Table 3:** Overall dimensions of foramina in each sex and their associations with the sex

	Mean±SD		
Foramen and its dimensions	Male (n=65×2)	Female (n=31×2)	*P* ^a^
Ovale			
Length (mm)	8.05±1.04	7.25±1.02	<0.001
Width (mm)	3.80±0.63	3.43±0.62	0.008
Area (mm^2^)	24.29±6.15	19.68±5.00	<0.001
Spinosum			
Length (mm)	2.49±0.30	2.15±0.36	<0.001
Width (mm)	2.00±0.30	1.83±0.25	0.007
Area (mm^2^)	3.95±0.90	3.14±0.81	<0.001
Rotundum			
Height (mm)	2.44±0.50	2.34±0.46	0.37
Width (mm)	2.44±0.56	2.31±0.53	0.29
Area (mm^2^)	4.72±1.55	4.29±1.32	0.18

^a^Independent sample *t*-test.

The width and area of FO showed a weak positive correlation with age (*r*=0.07 and 0.20, respectively), whereas the length of FO showed a weak negative correlation with age (*r*=−0.07). However, none of these correlations were statistically significant (*P*>0.05) (Table 4).

**Table 4:** Correlations of dimensions of the foramina with age (n=96×2)

	FO		FS		FR	
Variables	*r*	*P*	*r*	*P*	*r*	*P*
Length/height and age	−0.07	0.50	0.00	1.00	−0.09	0.34
Width and age	0.07	0.48	0.01	0.85	−0.03	0.77
Area and age	0.20	0.84	0.03	0.75	−0.05	0.57

FO, foramen ovale; FR, foramen rotundum; FS, foramen spinosum.

The width and area of FS showed a weak positive correlation with age (*r*=0.01 and 0.03, respectively), whereas the length of FS showed no correlation with age (*r*=0.00). However, none of these correlations were statistically significant (*P*>0.05) (Table 4).

Similarly, the height, width, and area of FR showed a weak negative correlation with age (*r*=−0.09, −0.03, and −0.05, respectively). However, none of these correlations were statistically significant (*P*>0.05) (Table 4).

The mean value was higher for length/height, width, and area of left FO, left FS, and left FR except for the higher mean value of the length of right FO. However, there was no statistically significant difference in right and left dimensions FO, FS, and FR (*P*>0.05) (Table 5).

**Table 5:** Dimensions of foramina of each side of body and their associations with laterality of the body

	Mean±SD			Minimum–maximum	
Foramen and its dimensions	Right (n=96)	Left (n=96)	*P* ^a^	Right (n=96)	Left (n=96)
Ovale					
Length (mm)	7.84±1.11	7.74±1.27	0.30	5.80–10.60	5.00–11.30
Width (mm)	3.65±0.77	3.72±0.69	0.31	1.90–5.60	2.00–5.30
Area (mm^2^)	22.65±6.55	22.95±7.06	0.61	8.80–44.40	11.12–41.69
Spinosum					
Length (mm)	2.35±0.40	2.41±0.45	0.17	1.10–3.10	1.20–3.60
Width (mm)	1.92±0.35	1.97±0.37	0.17	1.00–2.90	1.10–3.30
Area (mm^2^)	3.59±1.02	3.80±1.15	0.07	0.94–5.51	1.13–8.20
Rotundum					
Height (mm)	2.39±0.55	2.43±0.51	0.35	1.30–3.80	1.30–4.20
Width (mm)	2.39±0.60	2.41±0.58	0.69	1.10–4.30	1.00–3.80
Area (mm^2^)	4.54±1.66	4.62±1.52	0.07	1.21–10.46	1.26–9.25

^a^Paired sample *t*-test.

## Discussion

Adequate anatomical studies have been done where the foramina have been directly measured on cadaveric skull specimens^9^–^11^. However, only limited radiological studies with CT measurements of these foramina have been done^6^,^7^,^12^. Modern CT scanners are excellent in providing precise measurements of foramina which hardly differ from the direct measurements on anatomical specimens^4^,^6^,^7^. The observations of our study closely resembled previous studies. In the study by Berlis and colleagues, the CT measured mean (±SD) length and width of FO were 7.67±1.43 and 4.20±0.87 mm, respectively. In the same study, directly measured mean (±SD) length and width of FO on studied skull specimens were 7.41±1.31 and 3.91±0.77 mm, respectively.

The mean dimensions of both the FO and FS were significantly higher in males compared with females in our study. These observations closely resembled the results of study conducted by Berlis *et al.* 7 and Reymond *et al.* 13. However, in our study, there was statistically insignificant difference in the dimensions of FR between males and females despite higher mean values in male than female. The body habitus may account for higher dimensions in males than females^14^–^16^. There is a lack of literature comparing these dimensions in males and females and it requires further studies to draw some concrete conclusions.

In our study, none of the dimensions of the FO, FS, and FR showed significant correlations with the age of the participants. There is a lack of literature to establish this finding.

The dimensions of FO in our study resembled the various studies conducted on dry skulls in the Indian subcontinent. But the difference in dimensions of FO was not statistically significant in the current study, which is the same as multiple other studies, that is, Patil *et al.* 17, Ashwini and Venkateshu^18^, and Gupta and Rai^19^.

Moreover, it was also consistently found that the length, width, and area of FO have a wide range, for which the presence and the number of emissary veins passing through it can be assumed to be the main contributor^20^. Sepahdari and Mong^6^ reported easy reproducibility of the measurement of FO with high-resolution CT of the skull base and found greater variation between the subjects rather than that of left and right sides within the same subjects. Meanwhile, the study by Zdilla *et al.* 21, reported significant differences of major axes and area of paired left and right-sided FO with right dimensions more than left. The study by Ahmed *et al.* 22 concluded a significantly higher value of length and width of FO of the right side than the left side with a statistically insignificant difference in the area of FO of either side.

Dogan *et al.* 23, Kulkarni and Nikade^24^, and Reymond *et al.* 13 reported longitudinal and transverse diameters values of FS to be almost symmetrical on comparing right and left sides, with the insignificant difference in mean lengths and widths between the right and left FS. Another study by Emeka *et al.* 25 in Nigerian dry skulls, reported no significant difference between the mean length and width of the right and left FS, which is in accordance with the results of our study. In the current study, similar to several studies^23^–^25^ done in the past, there was no statistically significant difference between the length, width, and area of right and left FS. Apart from this, there could be variations in FO and FS as stated in a study of middle cranial fossa using CT scan, where the most common variation of FO and FS were oval shaped and a confluence of FS with FO, respectively^26^.

There was a statistically insignificant difference between the dimensions of right and left FR in the current study. The study by Dogan *et al.* 23 reported the longitudinal and transverse diameter values of right and left FR to be almost symmetrical and no statistical analysis was done for this. The study by Berge and Bergman^10^ reported FR to be consistently symmetrical, but when asymmetry existed, the left was more likely to be wider.

On review of the currently available literature, no plausible biological explanation could be found for the difference noted in this study. However, most of the previous studies were conducted by direct measurement among dry cadaveric skulls and there was a paucity of literature on studies conducted on objective CT measurements of these foramina. The current study also didn’t consider the body mass index of the participants, which could have potentially confounded our result, as males usually have a larger body habitus than females. Also, the representation of two sexes was not proportional in our study. This sex-based difference was a unique observation in this study and is an area with a dearth of literature. This could be a new area of interest and makes a firm scope for further radiological studies. In addition, we have not taken into account the inter-observer reliability which could also bias the findings.

## Conclusions

The mean length, width, and area of FO was 7.79±1.10 mm, 3.68±0.64 mm, and 22.80±6.18 mm^2^, respectively. The mean length, width, and area of FS was 2.38±0.36 mm, 1.94±0.30 mm, and 3.69±0.95 mm^2^, respectively. Similarly, the mean height, width, and area of FR was 2.41±0.49 mm, 2.40±0.55 mm, and 4.58±1.49 mm^2^, respectively. Although the dimensions of FR showed no significant sex-based difference, the dimensions of FO and FS were significantly higher in males compared with females. There was an insignificant difference in either of the dimensions of these foramina with age nor between the dimensions of right and left FO, FS, and FR. These findings would reinforce the existing database pertaining to the objective measurements of these foramina.

## Ethical approval

The ethical clearance was obtained from the institutional review committee of BPKIHS (Ref no: 199/076/077-IRC). Written informed consent was taken from each participant and the participation was entirely voluntary.

## Consent

Witten informed consent was obtained.

## Sources of funding

None.

## Authors’ contribution

R.B., P.K.S., S.K., M.B., M.K.G., and B.K. conceived and designed the study, with R.B. acting as the guarantor. R.B., S.P., G.K.Y., R.A., A.S., and B.K. performed the literature review. R.B., S.P., and G.K.Y. contributed to data collection and acquisition; performed the data, and statistical analysis. R.B., S.P., G.K.Y., and B.K. wrote the first draft of this manuscript. R.B., G.K.Y., R.A., A.S., and B.K. contributed to the final reviewing and editing of the manuscript before submission to the journal. R.B., S.B., P.K.S., S.K., M.B., M.K.G., and B.K. contributed to the critical revisions before approving the final draft of the manuscript for publication.

## Conflicts of interest disclosure

The authors declare that they have no financial conflict of interest with regard to the content of this report.

## Research registration unique identifying number (UIN)

1. Name of the registry: NA
2. Unique Identifying number or registration ID:
3. Hyperlink to your specific registration (must be publicly accessible and will be checked):

## Guarantor

Rakshya Bhattarai.

## Data availability statement

All the data pertaining to this article are present here within.

## Provenance and peer review

Not commissioned, externally peer reviewed.
